# Supplementary figures and images for: Targeting NPC1 in Renal Cell Carcinoma
Source: Cancers (Basel). 2024 Jan 25;16(3):517. doi: 10.3390/cancers16030517 (PMC10854724; doi:10.3390/cancers16030517)

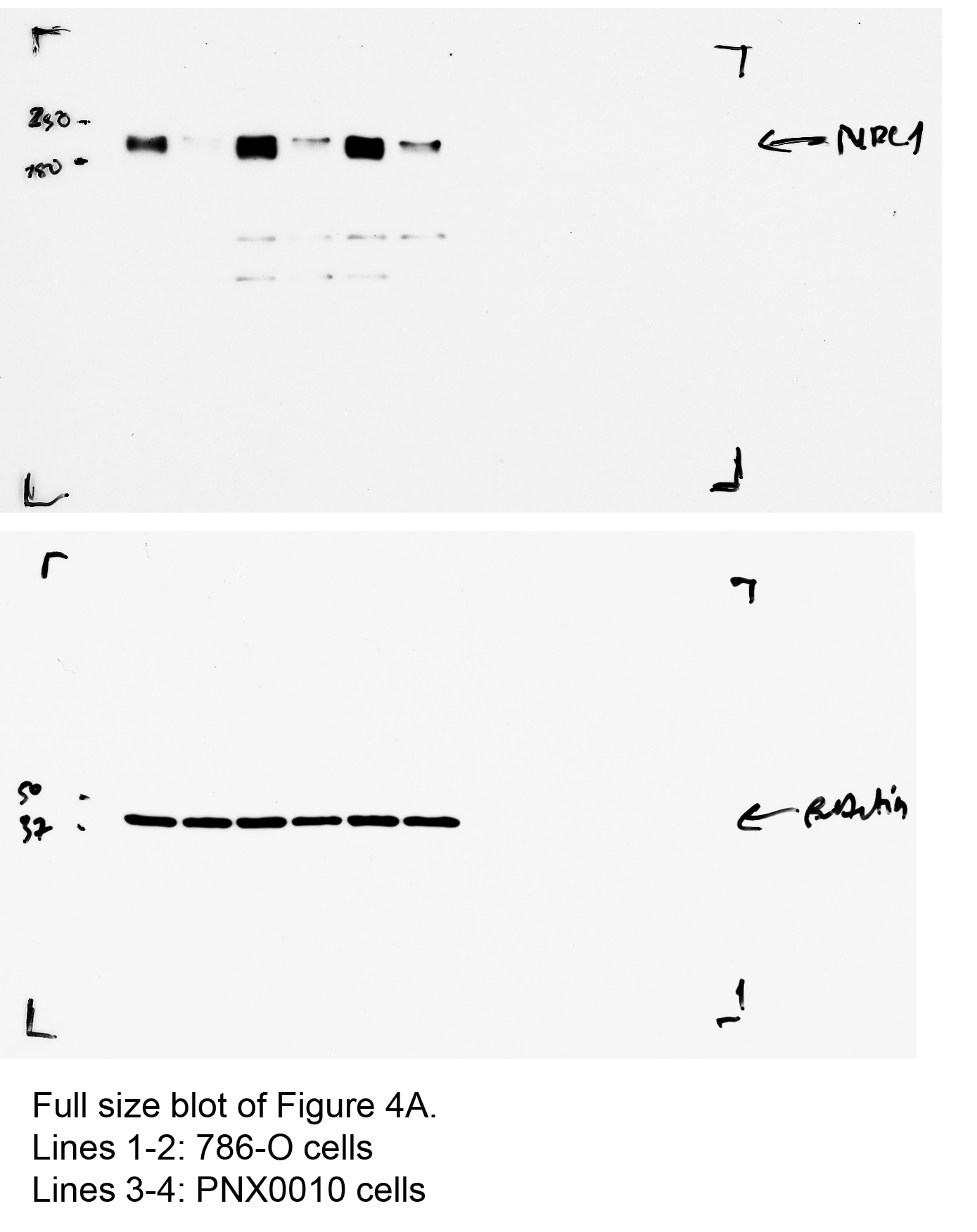

Supplement: Supplementary file 1 [file cancers-16-00517-s001.zip › cancers-2794469-supplementary.tif]
